# What works in sanitation promotion?

**DOI:** 10.1093/heapro/daad162

**Published:** 2023-12-06

**Authors:** Robert Aunger

**Affiliations:** Department for Disease Control, London School of Hygiene & Tropical Medicine, Keppel St., London, UK, WC1E 7HT

**Keywords:** sanitation, sanitation marketing, community development, community-led total sanitation, less-developed countries

## Abstract

Promotion appears to be the least effective but is nevertheless often the only available, means to achieve increased access to sanitation services, especially at scale, in lower-income countries. A cursory examination of the history of past and present approaches to sanitation promotion, including sanitation marketing, community development, community-led total sanitation and public health, shows that they have a variety of features and characteristics which make them distinctive. Unfortunately, rigorous evaluation has not kept pace with this proliferation of approaches, so it is difficult to recommend any one approach over the others, based on empirical performance in a range of circumstances. However, I argue that a ‘hybrid’ approach which exhibits a number of salient features from all of the previous approaches is likely to be a good bet. I present a recent example of such a hybrid programme which proved to significantly increase the rate of improved sanitation coverage through promotion (without subsidy of any kind) at scale in Tanzania. I suggest other sanitation promotion programs may want to think about adopting similar practices in their own programming going forward.

**Contribution to Health Promotion**

- Categorizes historical approaches to sanitation promotion
- Provides evidence for successful achievements of ‘hybrid’ approach
- Discusses features of ‘hybrid’ approach that could be used by future sanitation promotion programs

## INTRODUCTION

Sanitation, or the safe disposal of human faeces, is a fundamental necessity for the health of human societies (Giusti, 2009; Mara *et al.*, 2010; Freeman et al., 2017; Zhou *et al*., 2018). However, faeces management is often lacking at the household level in areas where families are impoverished, or where it is not seen as a priority—e.g. due to low population density (such as in rural areas). For this reason, agencies interested in public health have sought to increase sanitation coverage for a number of decades, and universal access to hygienic sanitation has become a global imperative with the inclusion of sanitation access in the Sustainable Development Goals. However, many countries are falling short of these goals as the deadlines for achieving universal access approach, lead many to wonder what can be done to speed up increases in the proportion of the population with access to hygienic sanitation (Black and Fawcett, 2008; Clarke, 2013).

Many different types of interventions have been tried over the years by government, donors and public health workers. A review that compared types of sanitation intervention found that incentive-based programs fared best in terms of increasing coverage, followed by education- or promotion-based programs (clumped together, although they are distinct in terms of psychological mechanisms), while community-led total sanitation (CLTS) fared least well (Garn *et al*., 2017). Similar results were shown by a more recent review, which showed that construction-based interventions worked best to increase sanitation coverage, followed by (mostly financial) incentives/subsidies, and then education/promotion (Igaki *et al.*, 2021). These are ­common-sensical results. It isn’t surprising that coverage increases most when facilities are simply provided, or, next-best, when the means for getting such facilities (i.e. money) are donated (or, more indirectly, the costs of getting such facilities are reduced—e.g. through the provision of new services), rather than trying to convince households to manage the task themselves. [Largely under the influence of behavioural economists, with their access to government officials, the idea of providing financial subsidies directly to households has been tried in numerous countries, mostly in recent decades (Bastagli *et al*., 2016; Ladhani and Sitter, 2020). However, since this is not a promotion, but a form of provision, it will not be addressed here, despite its popularity.] However, in many cases, governments or donors cannot afford provision at scale, especially to those living in areas where the provision of sanitation systems such as sewers is not economically or technologically feasible. Also, if managed poorly, or where there is no inherent demand, subsidies can lead to low usage, operational, maintenance and sustainability problems, not to mention the perception that something provided at low or no cost means the facility is of low value and hence not worth using (Whittington *et al*., 2012; Routray *et al*., 2015; Gupta *et al.*, 2019; Deep, 2022). Indirect incentives have also been tried in this sector, often in the form of introducing cheaper latrine slabs or container emptying services, but a profitable business model for novel sanitation services has not yet achieved scale, despite numerous attempts in many countries (Osterwalder and Pigneur, 2011; Larsen *et al*., 2015; Andersson *et al*., 2018; Baldi, 2019).

In the absence of these other strategies, countries are left with the least effective, but probably most often needed, means of increasing sanitation coverage: promotion (i.e. increasing the motivation for ­self-provision). The question then becomes how best to promote sanitation to households, especially in poor, low-density areas. Rural households’ demand for improved sanitation, at least, has historically been low (Jenkins and Sugden, 2006; O’Reilly and Louis, 2014).

This article presents a personal look at the past history of experiences with sanitation promotion, seeking to find a way to be more successful with such efforts in future. I conclude with an example of a recent ‘hybrid’ program that has been successful in achieving sanitation promotion at scale, without any subsidy, and suggest such a hybrid approach might be more effective in many circumstances (Aunger *et al*., 2023).

### A concise history of sanitation promotion

A number of approaches can be identified in the history of sanitation promotion since the 1990s. For the purposes of comparison, these approaches will be presented here as conceptual categories, since actual programs and projects tend to be mixtures of these approaches, implemented as complex interventions. [This categorization follows suggestions from the recent review by De Buck and colleagues (De Buck *et al*., 2017). Even earlier efforts to promote sanitation were largely based on educational programming, which has been ignored here, as it is no longer practised and was typically not very effective (Asaolu and Ofoezie, 2003; Emmett, 2006).] These categories are sanitation marketing, community development, CLTS, public health and a recent ‘hybrid’ approach. In the rest of this section, I first present each conceptual approach, discuss its history of implementation, then compare them at the conceptual level.

#### Sanitation marketing

An early approach taken by donors and NGOs was sanitation marketing (Cairncross, 2004; Jenkins and Scott, 2007; Devine and Kullmann, 2011; Sy and Warner, 2014). The assumption underlying this approach was that sanitation facilities or services can be promoted much like any other product—through marketing. This approach was sometimes called ‘social marketing’ because the benefits were seen to at least partly accrue to society rather than just those selling and purchasing the product or service (e.g. through health externalities). Nevertheless, the same techniques—of making sanitation seem desirable so that people would invest—were thought to apply.

However, the early popularity of sanitation marketing (prior to rigorous scientific interest in the Water, Sanitation and Hygiene [WASH] sector) and implementation mostly by NGOs meant that rigorous evaluations of this approach are almost entirely lacking (Evans *et al*., 2014; Nzioki and Korir, 2020). It may also have been the case that the most powerful techniques developed in the commercial sector were not applied, due to a lack of familiarity among those in the public sector with the highly guarded and often-secret marketing techniques developed by corporations, and requiring flexibility not suited to NGO funding cycles. In any case, the WASH sector largely turned its attention to other approaches.

#### Community development

An approach to which many turned was to facilitate the ability of communities to provide sanitation services for themselves, through some form of community development (Summers, 1986; Phillips and Pittman, 2008). This differs from the ‘do-it-yourself’ social marketing approach by targeting communities rather than sanitation ‘consumers’, but also by relying on means to increase capabilities at the community level that can be translated into increased sanitation coverage, rather than on marketing techniques. Typically there is a focus on community involvement and engagement, and shared decision-making within a novel organizational context, such as clubs or formalized groups (Hadi, 2000; Lenneiye, 2000), or with implementation by specialized community development teams (Mackenzie, 2008; Padawangi, 2010). Often, overlaying the effort is an explicit political agenda (Ledwith, 2020), frequently tied to notions of liberation and participatory action, although just as often, the concept of community development is criticized for being amorphous and abstract (Matarrita-Cascante and Brennan, 2012).

Women, in particular, who often experience greater burdens to well-being from lack of in-home sanitation, are often asked to take the lead in community development projects around sanitation (Pandya and Shukla, 2018). For example, microfinance groups can be introduced as a means of reducing risk and providing a quantity of money up-front (through pooling among households) that individual households may not be able to accumulate (Davis *et al.*, 2008; Mader, 2011). The introduction of microfinance institutions to support household-level investments in sanitation has produced spotty results and difficulties in going to scale (Geissler *et al*., 2016; Chunga *et al*., 2018). Other forms of novel group formation, such as women’s groups, have also been tried as a means to promoting sanitation (Waterkeyn and Cairncross, 2005; Waterkeyn and Waterkeyn, 2013), but again without proving to be a compelling model that has been widely adopted by others. As a consequence, community development is now continued, as a morally responsible aspect of sanitation promotion, but often combined with other approaches that might prove more effective.

#### Community-led total sanitation (CLTS)

CLTS (Kar and Chambers, 2008; Bongartz *et al.*, 2016) is given special attention here—even though it could be considered a form of community development, and is often seen as such by its advocates (Galvin, 2015; Lawrence *et al*., 2016; Zuin *et al*., 2019)—due to its degree of success. It differs from most community development efforts in its novel origins in the insights of a single person, and its reliance on conducting specific, inspirational events to spur interest in sanitation, rather than long-term ‘developmental’ efforts. It typically involves local government officials or their trained representatives moving around different communities running sanitation demand-creation events. These events include emotional demonstrations to mobilize communities, typically without direct subsidies for toilet construction. These demonstrations bring to light the fact that when communities do not dispose of their excreta safely, it can circulate socially and wind up in their diet (an insight often expressed in cruder terms). These demonstrations are meant to ‘trigger’ a community into a mutually agreed commitment to build proper sanitation facilities by a certain date. Should all local households do so, the community can be declared ‘open defecation free’ (Kar and Chambers, 2008).

Due to its perceived success, CLTS has become the official sanitation policy in many low-income countries—in fact, at least 60 countries to date (Zuin *et al*., 2019). However, initial improvements in coverage or use of improved toilets have often been followed by ‘slippage’ back into old patterns of behaviour; implementation can also vary considerably between countries, meaning that it isn’t the ‘same’ CLTS being evaluated everywhere (Haque and Freeman, 2021). Latrines also tend to be of lower quality than those built by households without such promotion, presumably because CLTS toilets are built simply to comply with group expectations and commitments (Perez, 2011; Crocker *et al*., 2017).

It is somewhat ironic that CLTS has proven so popular with national governments, given the lack of evidence for its sustainability, but this popularity seems to stem largely from advocacy by influential donors, NGOs and academics; robust scientific evidence has played little role in its diffusion (Zuin *et al*., 2019). Indeed, CLTS has not fared particularly well in formal evaluations (Venkataramanan *et al.*, 2018; Orgill-Meyer *et al*., 2019; Whittington *et al*., 2020). A recent global review was not sanguine about the ability of CLTS to produce sustained behaviour change, especially with respect to the building and use of *improved* sanitary facilities (USAID, 2018). Nevertheless, it remains the dominant approach at present for promoting household sanitation in low-income countries.

#### Public health

Public health projects are a rather different type of promotional exercise. These are largely conducted by health psychologists as a form of real-world testing of a theoretical notion, or empirical hypothesis, often about the psychological underpinnings of some phenomenon, like purchasing a household toilet. Many examples abound of this kind of approach, although few fall within the domain of sanitation, perhaps because sanitation is basically a service typically provided through the use of a relatively expensive product (a toilet at minimum) that is much harder—and more expensive—to promote than ‘lifestyle’ behaviours, for example.

Typically, such projects tend to be academic exercises—basically some form of demonstration, proof of concept study, or ideally a pilot for others to take up as an inspiring example (Mosler, 2012). They typically do analyses on sanitation-related data to address some hypothesis, such as the effectiveness of a theory-derived intervention (Tidwell *et al.*, 2019; Harter *et al*., 2020), or the relationship between some concepts and investment in sanitation (Tomberge *et al.*, 2022), or a study of the factors associated with prior investment in sanitation (Tumwebaze et al., 2013; Tobias *et al*., 2014), but can also piggyback on existing development projects (Mukhopadhyay, 2018), or re-analyse data from a new perspective (Liem *et al*., 2019). Since these tend to only be funded to the demonstration level, and due to the fact that academics rarely have good access to government officials or international donors, achieving scale with promising strategies has been a problem. Promoters have had to rely on scientific publication and rigorous evidence of the effectiveness of specific promotional strategies, to gain attention—and interest from major funders—to get implementation at larger scales.

#### Analysis

This quick history of approaches to sanitation promotion leaves a number of questions open—in particular, the question of which conceptual approach is empirically superior. Unfortunately, there haven’t been sufficient program evaluations or reviews to enable the isolation of particular features of a program that were responsible for its success or failure. So it isn’t possible at present to determine an ideal approach which includes the most effective components for getting households to invest in sanitation services in any or all circumstances. Systematic comparisons of the different promotional approaches have taken place, although many of the studies focus primarily on hand washing with soap rather than sanitation (De Buck *et al.*, 2017). The complexity of many sanitation interventions—which often combine subsidy or incentives with promotion—also makes the evaluation or comparison of approaches difficult (De Buck, 2017; Igaki *et al.*, 2021). It is therefore hard to conclude anything more profound than the ­common-sensical advice that making it easier for someone to get access to desirable/appealing/appropriate sanitation services is the best course of action.

Nevertheless, it is a relatively safe bet that an approach which takes advantage of many of the key features of the other approaches will have a better chance of being effective than one which utilizes only a few. Table 1 lists a number of features of the various approaches I have discussed. (It also has a column for a ‘hybrid’ approach that will be described later.) These features have been linked to the step in the program development process to which they are most fittingly attached. This process is described here in terms of the steps envisioned by behaviour-centred design (Aunger and Curtis, 2016; Aunger, 2020). These steps are: Assess (compiling what is already known about the causes of the target behaviour), Build (increasing relevant knowledge through original data collection exercises), Create (engaging in a process of translating research insights into motivational stimuli such as advertisements), Deliver (implementing activities that bring a campaign into contact with the target audience) and Evaluate (collecting and analysing data on the impact of the program implementation).

**Table 1:** Features associated with the different sanitation promotion approaches

Feature	Sanitation marketing	Community development	CLTS	Public health	‘Hybrid’
*Project management*					
Use of experts	X			X	X
Adaptive contracting					X
Private sector involvement	X				X
High-level government engagement		X	X		X
Reliance on a theory of change				X	X
*Assess phase*					
Use of experts				X	X
Contextual information survey				X	X
Coordination of stakeholders	X	X			X
*Build phase*		*NA*	*NA*		
Use of experts	X			X	X
Formative research	X			X	X
Insight generation	X			X	X
*Create phase*		*NA*	*NA*	*NA*	
Use of experts	X				X
Multiple prototype ideas generated	X				X
In-field testing of prototype ideas	X				X
Complex intervention development					X
*Delivery phase*					
Use of experts				X	X
Emphasis on training delivery personnel		X	X		
Real-time monitoring of outcomes/ impact	X				X
Emphasize messaging	X		X		X
Emphasize capacity- building		X			
Scale up reach via media	X				X
Scale up reach via implementer ‘army’		X	X		
Scale up reach via government activation			X		X
Emphasis on on-ground activations		X	X		X
Use of ‘emotional demonstrations’			X		X
Emphasis on education		X		X	
Reliance on subsidy		X			
Involvement of partners, including private sector					X
*Evaluation phase*	*NA*	*NA*	*NA*		
Use of experts				X	X
Conduct real-time evaluations					X
Conduct post-hoc evaluations				X	X
Disseminate findings for use by others				X	X

Inspection of the table shows that the approaches differ significantly in terms of the features, or active components, they exhibit. For example, only sanitation marketing is likely to involve private sector players or monitor outcomes; only community development emphasizes capacity development, while only public health relies on a psychologically explicit theory of change. Most significantly, some approaches skip entire steps in the program development process, such as engaging in on-the-ground research to learn of local conditions and circumstances (community development and CLTS), or involving an extensive creative period to translate perceived insights into motivational campaign materials (all approaches except sanitation marketing) or engage in the scientifically rigorous evaluation of program outcomes (all except public health).

Many of these features arise as a function of certain characteristics of each approach. These distinctive characteristics are captured in Table 2, which shows that the approaches have different primary actors, funding sources, patterns of expenditure, heydays, tactics, objectives, kinds of effects on households and means to achieve scale. The characteristics of an approach can be linked to its features. For example, public health approaches rely on theories of change (a feature) because they are typically devised by scientists such as health psychologists with an interest in understanding the mechanisms underlying outcomes from the testing of some hypothesis (a characteristic). Similarly, community development programs involve training community members to organize to deliver outcomes (a feature), so training becomes their primary kind of financial expenditure (a characteristic). CLTS is almost alone in emphasizing on-ground activations (a few sanitation marketing and community development programs have included direct consumer contact events), often involving emotional demonstrations to create a shared insight about the consequences of their common practices (features)—and so depends on repeated events to achieve scale (a characteristic). These significant differences help explain why they can be distinguished from each other conceptually, despite the overlaps in actual sanitation programming. They are quite different ways of trying to promote investment by households in sanitation, presumably with mixed degrees of success.

**Table 2:** Characteristics of sanitation promotion approaches

Feature	Community development	Sanitation marketing/Promotion	CLTS	Public health	‘Hybrid’
Primary actor	Government	NGO	Government	University	Consortium including government
Funding	NGOs	NGOs	Government	Science funders	Various
Heyday	1990s	2000s	2005–2015	2000-	2015-
Objective	Government 5-year plan	Demonstration project goals	Government 5-year plan	Demonstration project goals	Various
Promotional tactic	Community involvement	Marketing techniques	Emotional demonstrations (‘Emo-demos’)	Motivational enhancement	Exposure to creative messaging; on-ground activations
Primary expenditure	Training	Media	Training/ Personnel	Project personnel salaries	Program activities
Effect at household level	Potential program involvement	Increased motivation	Commitment to community goal	Various	Increased motivation
Role of government	Implementer	None	Implementer	None	Secondary target audience/ Project manager
Means to scale	Implementation activities	Uptake by other actors	Repeated action	Adoption by governments	Uptake by other actors
Examples	SHEWA-B (Huda *et al.*, 2012)	WSP Scaling Up Rural Sanitation project (Perez et al., 2012)	Various national programs	WASH benefits and Shine trials (Pickering *et al.*, 2019)	*Nyumba ni choo* (Tanzania) (Aunger *et al.*, 2023)

### Going forward: A ‘hybrid’ approach

The foregoing analysis suggests that an approach which combines the elements from previous approaches might be the best way forward for the WASH sector. This ‘hybrid’ approach could thus capitalize on the accumulated knowledge from prior experience, evaluations and reviews to design a more effective approach.

An example of a recent program that adopted ‘hybrid’ methods for project development, management and implementation was Tanzania’s National Sanitation Campaign ‘*Nyumba ni choo*’ (which roughly translates as ‘a house is not complete without a proper toilet’) (Aunger *et al*., 2023). Funded by DFID/FCDO, the UK government’s international development agency, led by the Tanzania Ministry of Health, and with services provided by a consortium composed of representatives from a university faculty, creative agencies, and implementation and management companies, this program achieved considerable success at getting households to invest in their own sanitation facilities, without subsidy, and at a national scale. Implementation involved a diverse set of intervention components:

- standard scripted advertisements
- ‘reality-TV’ shows involving toilet makeovers
- entertaining ‘roadshows’ put on by a troupe of acrobats and actors led by a nationally famous singer (including ‘emo-demos’ or emotional demonstrations—activities such as group commitments—to motivate investment in sanitation and hygiene)
- mentions during regular chat shows on regional radio
- business management events for entrepreneurs in the WASH sector
- social media postings about these various program activities

This program shares many of the features of prior approaches (see Table 1), while also adopting a number of features not previously seen in the sector—primarily associated with modern management practices, such as adaptive programming (Derbyshire and Donovan, 2016; Valters *et al*., 2016). Adaptive programming, generally speaking, is a flexible style of management that dynamically evolves in response to changing conditions on the ground, even to the point of modifying its objectives in light of evidence of implementation failures, or new directives from stakeholders. At the same time, the hybrid approach is quite distinctive compared to the other approaches in terms of its characteristics (see Table 2). For example, creative materials were constantly refreshed, partner organizations in the management coalition were swapped in and out depending on current needs, and conditions on the ground were continually monitored through bespoke surveys.

The Ministry of Health, through their National Sanitation Management Information System, report that during this program (between 2017 and 2021) the level of improved sanitation coverage in Tanzania nearly doubled, from 43.5% to 71.4% of households, showing a considerably increased rate of improvement over the previous trend (Aunger, submitted). The campaign even became a platform the government used to promote other health behaviours (e.g. COVID-19 transmission prevention).

The hybrid approach differs from the others in significant ways which probably contribute to its success:

- It has both media and on-the-ground activations (which were then used as content for media creation).
- Target audiences are potentially hit numerous times via various modes of contact.
- Practitioners conducted real-time evaluations to modify creative content during implementation (to reflect changing circumstances and behavioural goals).
- The management team engaged in adaptive contracting and flexible team membership.
- Government buy-in, at both national and local levels, meant there was support throughout the country for the program.
- Private sector providers of sanitation products were included in promotional events.

The hybrid-based campaign became a widely recognized ‘brand’ which changed public perceptions about the nature of sanitation (it too can be part of a modern lifestyle and a feature of public discourse). The program was also embedded in the government’s agenda (the government was an active participant in the consortium), which meant that they sought out additional funding from other sources as a priority to maintain the program’s impetus and momentum. Furthermore, the consortium’s local leadership incorporated a new profit-making business that also sought new clients to continue the good work it had already done.

## Conclusion

This brief review of the history of sanitation promotion suggests that we *can* learn lessons from the past through a clear-headed analysis of the evidence and comparison of different approaches. Whilst it is probably fair to say that each approach reviewed here—sanitation marketing, community development, CLTS and public health programming—can lead to success in particular circumstances, in many cases, sanitation promotional programs have failed at their objectives. It isn’t possible to make a more pointed recommendation due to the lack of definitive evidence for or against particular implementation features. However, it might make sense to take advantage of a hybrid approach which combines elements from others to increase the probability of success. I have described a recent campaign which used such a hybrid approach and was successful at promoting household-level investment in improved sanitation, at the national scale, in a low- or middle-income country, Tanzania. It combined a number of features from the other approaches, together with adaptive programming-based management, which allows a program—and its outcomes—to be sustained in the face of changing circumstances, even over the long term. This is obviously just one example, so no definitive conclusions can be drawn. Nevertheless, given the fundamental need for hygienic sanitation around the world, and the likelihood that many countries will fall short of their Sustainable Development Goal for access to sanitation, new options need to be explored. Using a ‘hybrid’ approach, with dynamic adaptation to changing on-ground circumstances, could be a way forward for the sector.

## References

[CIT0001] Andersson, K., Otoo, M. and Nolasco, M. (2018) Innovative sanitation approaches could address multiple development challenges. Water Science and Technology, 77, 855–858. 29488947 10.2166/wst.2017.600

[CIT0002] Asaolu, S. O. and Ofoezie, I. E. (2003) The role of health education and sanitation in the control of helminth infections. Acta Tropica, 86, 283–294. 12745145 10.1016/s0001-706x(03)00060-3

[CIT0003] Aunger, R. (2020) Reset: An Introduction to Behaviour Centred Design. Oxford University Press, Oxford.

[CIT0004] Aunger, R. and Curtis, V. (2016) Behaviour centred design: towards an applied science of behaviour change. Health Psychology Review, 10, 425–446. 27535821 10.1080/17437199.2016.1219673 PMC5214166

[CIT0005] Aunger, R., Mwambuli, K. and Cardosi, J. (2023) Lessons from a successful national sanitation programme: the case of Nyumba ni Choo in Tanzania. Health Promotion International, 38, daad126. 37815063 10.1093/heapro/daad126 PMC10563015

[CIT0006] Baldi, M. (2019) Sanitation solutions: business models for access. Gates Open Research, 3, 1674.

[CIT0007] Bastagli, F., Hagen-Zanker, J. and Harman, L. (2016) Cash transfers: what does the evidence say. A rigorous review of programme impact and of the role of design and implementation features. Technical Report. Overseas Development Institute.

[CIT0008] Black, M. and Fawcett, B. (2008) The Last Taboo: Opening the Door on the Global Sanitation Crisis. Routledge, London.

[CIT0009] Bongartz, P., Vernon, N. and Fox, J. (2016) Sustainable Sanitation for All: Experiences, Challenges, and Innovations. Practical Action Publishing, Rugby, England.

[CIT0010] Cairncross, S. (2004) The case for marketing sanitation. Water & Sanitation Program Field Note. Technical Report. The World Bank, Nairobi.

[CIT0011] Chunga, R., Jenkins, M. W., Ensink, J. and Brown, J. (2018) Moving up the sanitation ladder with the help of microfinance in urban Malawi. Journal of Water, Sanitation and Hygiene for Development, 8, 100–112.

[CIT0012] Clarke, R. (2013) Water: The International Crisis. Routledge, London.

[CIT0013] Crocker, J., Saywell, D. and Bartram, J. (2017) Sustainability of community-led total sanitation outcomes: evidence from Ethiopia and Ghana. International Journal of Hygiene and Environmental Health, 220, 551–557. 28522255 10.1016/j.ijheh.2017.02.011 PMC5475437

[CIT0014] Davis, J., White, G., Damodaron, S. and Thorsten, R. (2008) Improving access to water supply and sanitation in urban India: microfinance for water and sanitation infrastructure development. Water Science and Technology, 58, 887–891. 18776626 10.2166/wst.2008.671

[CIT0015] De Buck, E. et al . (2017) Promoting handwashing and sanitation behaviour change in low-and middle-income countries: a mixed-method systematic review. *3ie Systematic Review*, 36.

[CIT0016] Deep, P. (2022) Public provisioning for making India open defecation free and the implication of Swachh Bharat mission (Clean India Campaign). International Journal of Food and Nutritional Sciences, 11(7):799–815.

[CIT0017] Derbyshire, H. and Donovan, E. (2016) *Adaptive Programming in Practice: Shared Lessons from the DFID-funded LASER and SAVI Programmes*. London: DFID.

[CIT0018] Devine, J. and Kullmann, C. (2011) *Introductory Guide to Sanitation Marketing. Water and Sanitation Program Toolkit*. World Bank, Washington, DC.

[CIT0019] Emmett, B. (2006) In the Public Interest: Health, Education, and Water and Sanitation for All. Oxfam, London.

[CIT0020] Evans, W. D., Pattanayak, S. K., Young, S., Buszin, J., Rai, S. and Bihm, J. W. (2014) Social marketing of water and sanitation products: a systematic review of peer-reviewed literature. Social Science and Medicine, 110, 18–25. 24704890 10.1016/j.socscimed.2014.03.011

[CIT0021] Freeman, M. C., Garn, J. V., Sclar, G. D., Boisson, S., Medlicott, K., Alexander, K. T. et al. (2017) The impact of sanitation on infectious disease and nutritional status: a systematic review and meta-analysis. International Journal of Hygiene and Environmental Health, 220, 928–949. 28602619 10.1016/j.ijheh.2017.05.007

[CIT0022] Galvin, M. (2015) Talking shit: is community-led total sanitation a radical and revolutionary approach to sanitation? Wiley Interdisciplinary Reviews: Water, 2, 9–20.

[CIT0023] Garn, J. V., Sclar, G. D., Freeman, M. C., Penakalapati, G., Alexander, K. T., Brooks, P. et al. (2017) The impact of sanitation interventions on latrine coverage and latrine use: a systematic review and meta-analysis. International Journal of Hygiene and Environmental Health, 220, 329–340. 27825597 10.1016/j.ijheh.2016.10.001 PMC5414716

[CIT0024] Geissler, K. H., Goldberg, J. and Leatherman, S. (2016) Using microfinance to facilitate household investment in sanitation in rural Cambodia. Health Policy and Planning, 31, 1193–1199. 27198981 10.1093/heapol/czw051

[CIT0025] Giusti, L. (2009) A review of waste management practices and their impact on human health. Waste Management, 29, 2227–2239. 19401266 10.1016/j.wasman.2009.03.028

[CIT0026] Gupta, A. et al . (2019) *Coercion, Construction, and ‘ODF Paper Pe’: Swachh Bharat According to Local Officials*. SocArXiv c3va8, Center for Open Science.

[CIT0027] Hadi, A. (2000) A participatory approach to sanitation: experience of Bangladeshi NGOs. Health Policy and Planning, 15, 332–337. 11012409 10.1093/heapol/15.3.332

[CIT0028] Haque, S. S. and Freeman, M. C. (2021) The applications of implementation science in water, sanitation, and hygiene (WASH) research and practice. Environmental Health Perspectives, 129, 65002. 34132602 10.1289/EHP7762 PMC8207965

[CIT0029] Harter, M., Inauen, J. and Mosler, H. J. (2020) How does ­community-led total sanitation (CLTS) promote latrine construction, and can it be improved? A cluster-randomized controlled trial in Ghana. Social Science and Medicine, 245, 112705. 31838334 10.1016/j.socscimed.2019.112705 PMC6983942

[CIT0070] Huda, T.M. et al. (2012) Interim evaluation of a large scale sanitation, hygiene and water improvement programme on childhood diarrhea and respiratory disease in rural Bangladesh. *Soc Sci Med*, 75, 604–611 22197292 10.1016/j.socscimed.2011.10.042

[CIT0030] Igaki, S., Duc, N. T. M., Nam, N. H., Nga, T. T. T., Bhandari, P., Elhamamsy, A. et al. (2021) Effectiveness of community and school-based sanitation interventions in improving latrine coverage: a systematic review and meta-analysis of randomized controlled interventions. Environmental Health and Preventive Medicine, 26, 1–12. 33627071 10.1186/s12199-021-00934-4 PMC7903680

[CIT0031] Jenkins, M. W. and Scott, B. (2007) Behavioral indicators of household decision-making and demand for sanitation and potential gains from social marketing in Ghana. Social Science and Medicine, 64, 2427–2442. 17442472 10.1016/j.socscimed.2007.03.010

[CIT0032] Jenkins, M. W. and Sugden, S. (2006) *Rethinking Sanitation: Lessons and Innovation for Sustainability and Success in the New Millennium*. Human Development Report Office (HDRO), United Nations Development Programme.

[CIT0033] Kar, K. and Chambers, R. (2008) Handbook on Community-Led Total Sanitation. Plan UK, London.

[CIT0034] Ladhani, S. and Sitter, K. C. (2020) Conditional cash transfers: a critical review. Development Policy Review, 38(1), 28–41.

[CIT0035] Larsen, T. A., Gebauer, H., Gründl, H., Künzle, R., Lüthi, C., Messmer, U. et al. (2015) Blue diversion: a new approach to sanitation in informal settlements. Journal of Water, Sanitation and Hygiene for Development, 5, 64–71.

[CIT0036] Lawrence, J. J., Yeboah-Antwi, K., Biemba, G., Ram, P. K., Osbert, N., Sabin, L. L. et al. (2016) Beliefs, behaviors, and perceptions of community-led total sanitation and their relation to improved sanitation in rural Zambia. American Journal of Tropical Medicine and Hygiene, 94, 553–562. 26787149 10.4269/ajtmh.15-0335 PMC4775890

[CIT0037] Ledwith, M. (2020) Community Development: A Critical Approach. Policy Press, Bristol.

[CIT0038] Lenneiye, M. (2000) Testing community empowerment strategies in Zimbabwe: examples from nutrition supplementation, and water supply and sanitation programmes. IDS Bulletin, 31, 21–29.

[CIT0039] Liem, S., Marta, R. F. and Panggabean, H. (2019) Sanitation behavior and risk of stunting: understanding the discourse of a public service announcement. Jurnal The Messenger, 11(2).

[CIT0040] Mackenzie, U. (2008) A Tale of Two CDs: Capacity Development and Community Development in the Waste, Water, and Sanitation Sector in Kiribati. Asian Development Bank, Mandaluyong City, Phil.

[CIT0041] Mader, P. (2011) Attempting the production of public goods through microfinance: the case of water and sanitation. Journal of Infrastructure Development, 3, 153–170.

[CIT0042] Mara, D., Lane, J., Scott, B. and Trouba, D. (2010) Sanitation and health. PLoS Medicine, 7, e1000363. 21125018 10.1371/journal.pmed.1000363 PMC2981586

[CIT0043] Matarrita-Cascante, D. and Brennan, M. A. (2012) Conceptualizing community development in the ­twenty-first century. Community Development, 43, 293–305.

[CIT0044] Mosler, H. -J. (2012) A systematic approach to behavior change interventions for the water and sanitation sector in developing countries: a conceptual model, a review, and a guideline. International Journal of Environmental Health Research, 22, 431–449. 22292899 10.1080/09603123.2011.650156

[CIT0045] Mukhopadhyay, B. (2018) *Network Externalities with Sanitation: The Case of Swaach Bharat Mission*.

[CIT0046] Nzioki, J. M. and Korir, A. (2020) Effective methods for community sanitation and hygiene promotion in the developing world: a scoping review. Africa Journal of Technical and Vocational Education and Training, 5, 175–185.

[CIT0047] O’Reilly, K. and Louis, E. (2014) The toilet tripod: understanding successful sanitation in rural India. Health Place, 29, 43–51. 24954614 10.1016/j.healthplace.2014.05.007

[CIT0048] Orgill-Meyer, J., Pattanayak, S. K., Chindarkar, N., Dickinson, K. L., Panda, U., Rai, S. et al. (2019) Long-term impact of a community-led sanitation campaign in India, 2005–2016. Bulletin of the World Health Organization, 97, 523–533A. 31384071 10.2471/BLT.18.221572 PMC6653825

[CIT0049] Osterwalder, A. and Pigneur, Y. (2011) Aligning profit and purpose through business model innovation. In Palazzo, G. and Wentland, M. (eds), Responsible Management Practices for the 21st Century. Pearson International, Cambridge pp. 61–76.

[CIT0050] Padawangi, R. (2010) Community-driven development as a driver of change: water supply and sanitation projects in rural Punjab, Pakistan. Water Policy, 12, 104–120.

[CIT0051] Pandya, M. N. and Shukla, P. S. (2018) Role of women led sanitation in community development. Journal of Content, Community and Communication, 4, 71–77.

[CIT0071] Perez, E. A. et al. (2012) What does it take to scale up rural sanitation? World Bank, Washington, DC.

[CIT0052] Perez, E. A. (2011) *Sustainable Rural Sanitation at Scale: Results and Lessons from India, Indonesia, and Tanzania*. Loughborough University.

[CIT0072] Pickering, A.J. et al. (2019) The WASH Benefits and SHINE trials: interpretation of WASH intervention effects on linear growth and diarrhoea. *The Lancet Global Health*, 7, e1139–e1146. 31303300 10.1016/S2214-109X(19)30268-2

[CIT0053] Phillips, R. and Pittman, R. (2008) An Introduction to Community Development. Routledge, London.

[CIT0054] Routray, P., Schmidt, W. -P., Boisson, S., Clasen, T. and Jenkins, M. W. (2015) Socio-cultural and behavioural factors constraining latrine adoption in rural coastal Odisha: an exploratory qualitative study. BMC Public Health, 15, 880. 26357958 10.1186/s12889-015-2206-3 PMC4566293

[CIT0055] Summers, G. F. (1986) Rural community development. Annual Review of Sociology, 12, 347–371.

[CIT0056] Sy, J. and Warner, R. (2014) *Tapping the Markets: Opportunities for Domestic Investments in Water and Sanitation for the Poor*.

[CIT0057] Tidwell, J. B., Chipungu, J., Bosomprah, S., Aunger, R., Curtis, V. and Chilengi, R. (2019) Effect of a behaviour change intervention on the quality of peri-urban sanitation in Lusaka, Zambia: a randomised controlled trial. The Lancet Planetary Health, 3, e187–e196. 31029230 10.1016/S2542-5196(19)30036-1

[CIT0058] Tobias, R., Meyer, P. and O’Keefe, M. (2014) Investigating slum dwellers decisions to invest in a sanitation service in Kampala (Uganda). European Health Psychologist, 16.

[CIT0059] Tomberge, V. M. J., Harter, M. and Inauen, J. (2022) The importance of collective and individual psychological ownership for safe sanitation: a multilevel analysis in rural Ghana. Global Public Health, 17, 1314–1329. 34016017 10.1080/17441692.2021.1928260

[CIT0060] Tumwebaze, I. K., Orach, C. G., Niwagaba, C., Luthi, C. and Mosler, H. -J. (2013) Sanitation facilities in Kampala slums, Uganda: users’ satisfaction and determinant factors. International Journal of Environmental Health Research, 23, 191–204. 22873693 10.1080/09603123.2012.713095

[CIT0061] USAID (2018) *An Examination of CLTS’s Contributions toward Universal Sanitation*.

[CIT0062] Valters, C., Cummings, C. and Nixon, H. (2016) *Putting Learning at the Centre: Adaptive Development Programming in Practice*.

[CIT0063] Venkataramanan, V., Crocker, J., Karon, A. and Bartram, J. (2018) Community-led total sanitation: a mixed-methods systematic review of evidence and its quality. Environmental Health Perspectives, 126, 026001. 29398655 10.1289/EHP1965 PMC6066338

[CIT0064] Waterkeyn, J. and Cairncross, S. (2005) Creating demand for sanitation and hygiene through community health clubs: a cost-effective intervention in two districts in Zimbabwe. Social Science and Medicine, 61, 1958–1970. 15927329 10.1016/j.socscimed.2005.04.012

[CIT0065] Waterkeyn, J. A. and Waterkeyn, A. J. (2013) Creating a culture of health: hygiene behaviour change in community health clubs through knowledge and positive peer pressure. Journal of Water, Sanitation and Hygiene for Development, 3, 144.

[CIT0066] Whittington, D., Jeuland, M., Barker, K. and Yuen, Y. (2012) Setting priorities, targeting subsidies among water, sanitation, and preventive health interventions in developing countries. World Development, 40, 1546–1568.

[CIT0067] Whittington, D., Radin, M. and Jeuland, M. (2020) Evidence-based policy analysis? The strange case of the randomized controlled trials of community-led total sanitation. Oxford Review of Economic Policy, 36, 191–221.

[CIT0068] Zhou, X., Li, Z., Zheng, T., Yan, Y., Li, P., Odey, E. A. et al. (2018) Review of global sanitation development. Environment International, 120, 246–261. 30103124 10.1016/j.envint.2018.07.047 PMC6192828

[CIT0069] Zuin, V., Delaire, C., Peletz, R., Cock-Esteb, A., Khush, R. and Albert, J. (2019) Policy diffusion in the rural sanitation sector: lessons from Community-Led Total Sanitation (CLTS). World Development, 124, 104643.

